# Characterization of *TM8*, a MADS-box gene expressed in tomato flowers

**DOI:** 10.1186/s12870-014-0319-y

**Published:** 2014-11-30

**Authors:** Margherita Daminato, Simona Masiero, Francesca Resentini, Alessandro Lovisetto, Giorgio Casadoro

**Affiliations:** Department of Biology, University of Padua, Via G. Colombo, 3, 35131 Padua, Italy; Department of Bioscience, University of Milan, Via Celoria, 26, 20133 Milan, Italy; Botanical Garden, University of Padua, Via Orto Botanico, 15, 35123 Padua, Italy

**Keywords:** MADS-box genes, *Solanum lycopersicon*, *TM8* gene, TM8 protein interactions, Tomato flower development, Two-hybrid assays

## Abstract

**Background:**

The identity of flower organs is specified by various MIKC MADS-box transcription factors which act in a combinatorial manner. *TM8* is a MADS-box gene that was isolated from the floral meristem of a tomato mutant more than twenty years ago, but is still poorly known from a functional point of view in spite of being present in both Angiosperms and Gymnosperms, with some species harbouring more than one copy of the gene. This study reports a characterization of *TM8* that was carried out in transgenic tomato plants with altered expression of the gene.

**Results:**

Tomato plants over-expressing either *TM8* or a chimeric repressor form of the gene (*TM8:SRDX*) were prepared. In the *TM8* up-regulated plants it was possible to observe anomalous stamens with poorly viable pollen and altered expression of several floral identity genes, among them B-, C- and E-function ones, while no apparent morphological modifications were visible in the other whorls. Oblong ovaries and fruits, that were also parthenocarpic, were obtained in the plants expressing the *TM8:SRDX* repressor gene. Such ovaries showed modified expression of various carpel-related genes. No apparent modifications could be seen in the other flower whorls. The latter plants had also epinastic leaves and malformed flower abscission zones. By using yeast two hybrid assays it was possible to show that TM8 was able to interact in yeast with MACROCALIX.

**Conclusions:**

The impact of the ectopically altered *TM8* expression on the reproductive structures suggests that this gene plays some role in the development of the tomato flower. *MACROCALYX*, a putative A-function MADS-box gene, was expressed in all the four whorls of fully developed flowers, and showed quantitative variations that were opposite to those of *TM8* in the anomalous stamens and ovaries. Since the TM8 protein interacted *in vitro* only with the A-function MADS-box protein MACROCALYX, it seems that for the correct differentiation of the tomato reproductive structures possible interactions between TM8 and MACROCALYX proteins might be important.

**Electronic supplementary material:**

The online version of this article (doi:10.1186/s12870-014-0319-y) contains supplementary material, which is available to authorized users.

## Background

The availability of floral homeotic mutants and the isolation of the corresponding genes represented a turning point in our understanding of the molecular basis of flower formation. These studies were mostly done in *Arabidopsis thaliana*, *Antirrhinum majus* and *Petunia hybrida*, and led to the characterization of various MADS-box transcription factors that were shown to be able to switch on genetic programs leading to the actual formation of the flower organs [1,2].

Tomato (*Solanum lycopersicon*) is a species of enormous and worldwide economic importance and a large collection of mutants is presently hosted at the Tomato Genetics Resource Center (http://tgrc.ucdavis.edu). Unfortunately, no tomato floral homeotic mutants were available in 1990’, when the ABC model was proposed, therefore in those years efforts were focused on the isolation of tomato MADS-box genes by screening with heterologous probes two tomato cDNA libraries prepared from mRNA of mature wild type flowers and *anantha* floral meristems, respectively [3]. In the *anantha* mutant the floral meristems are blocked before formation of the flower and they branch indefinitely giving rise to a cauliflower-like inflorescence [4].

Several MADS-box coding cDNAs were thus isolated and named *TM* (*Tomato MADS*) followed by a number. In particular, from the arrested floral meristem of the *anantha* mutant it was isolated a gene that was named *TM8*, and was regarded as an “early” gene along the pathway of flower formation together with *TM4*, while *TM5*, *TM6* and *TM16* were regarded as “late” genes along the same pathway [3]. Northern blot assays revealed that *TM8* was expressed in pistils, anthers and petals, although at much lower levels than the other MADS-box genes. No transcripts were detected in sepals and leaves [3] but this result might have depended on the low sensitivity of the Northern technique compared to more modern types of analysis.

In a comprehensive work aimed at the characterization of the MADS-box gene family in tomato, Hileman *et al*. [5] isolated a large number of new genes, and analyzed by semi-quantitative RT-PCR their expression together with that of the previously known genes. It was thus shown that *TM8* is expressed not only in the reproductive apparatus, but also in leaves.

Probably the absence of a TM8 ortholog in Arabidopsis made this gene of poor interest for further characterization. However, the limited knowledge of the possible function(s) performed by *TM8* was recently stressed by Heijmans *et al*. [6] who suggested that its functional characterization might be “of special interest in order to complete our understanding of MIKC^c^ gene function”.

Matter of fact, after having isolated the first tomato MADS-box genes, the same research group tried to functionally characterize them by preparing transgenic anti-sense plants for each gene. However, to our best knowledge, the only reference to the results obtained with *TM8* is that found in Lifschitz *et al*. [7] where it was reported that 3 out of 12 transgenic plants exhibited severe deformation of the ovary and complete sterility. Also an extremely high incidence of parthenocarpy was reported.

Since after more than 20 years from the discovery of the *TM8* gene the information about its function is still very scarce, we deemed of interest to study the possible role played by this gene in tomato. To do so we prepared transgenic tomato plants over-expressing it, but also transgenic plants over-expressing a chimeric gene carrying the *TM8* sequence fused to the SRDX transcriptional repressor domain [8,9]. The results obtained in this work indicate that TM8 may be important for anther but also for ovary and fruit formation. Moreover, some phenotypic alterations were observed also for the leaves and this is in accordance with the observed normal expression of *TM8* in this organ.

## Results

A functional characterization of *TM8* was carried out by preparing transgenic plants with altered expression of the gene. Its related cDNA was obtained by RT-PCR experiments using specific oligonucleotides designed on the X60760 sequence [3]. The cDNA thus obtained was sequenced on both strands and it appeared that its coding region was actually 60 nucleotides longer than that of the X60760 sequence. In particular, 42 and 18 extra-nucleotides were localized in the K and C domains, respectively (not shown). Specific primers able to discriminate between the X60760 sequence and the *TM8* sequence isolated by us were used for PCR experiments with both genomic DNA and cDNA obtained from flowers and fruits. The results (Additional file 1) showed that both sequences are present in the tomato genome, and this is in agreement with data from the published genome [10]. However, only the longer sequence isolated by us appeared to be expressed in the flowers and fruits of our tomato plants (i.e. cv Florida Petite), therefore we decided to use the latter cDNA for the preparation of transgenic Florida Petite plants.

### Phenotypic characterization of plants over-expressing the *TM8* gene

**Figure 1:**
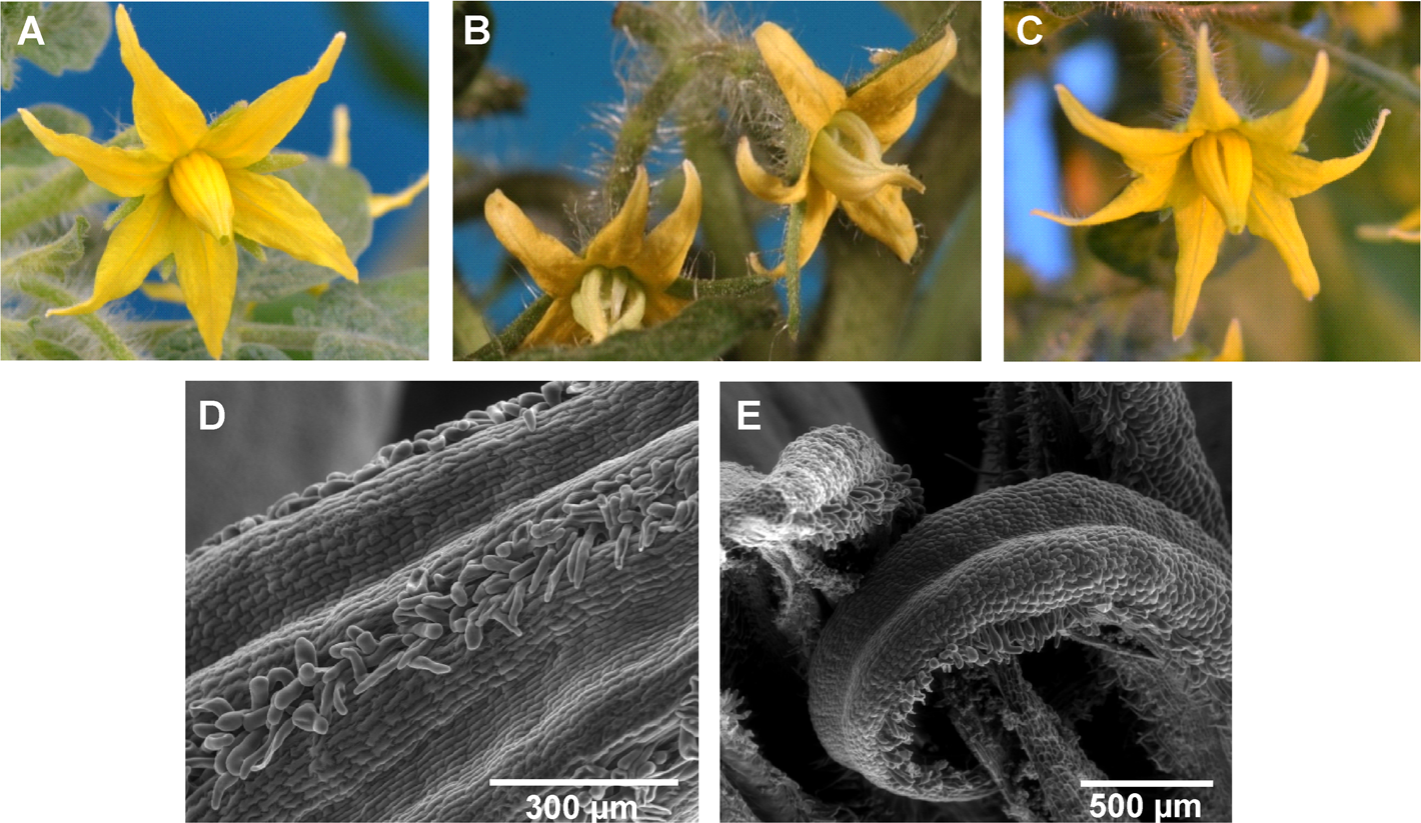
***35S:TM8*** **plant phenotype.** Wild-type tomato flower **(A)** and flowers of the lines *35S:TM8*#16 and *35S:TM8*#11 **(B,C)** over-expressing the *TM8* gene and showing splayed out stamens. ESEM (environmental scanning electron microscopy) pictures of a wild-type staminal cone **(D)** showing the interweaving hairs of the adjoining anthers and a transgenic splayed out cone of the line *35S:TM8*#16 **(E)** showing an anther not joined to others.

22 independent lines were obtained that harbored the *35S:TM8* construct as determined by PCR analyses carried out with genomic DNA extracted from leaves (see Methods - generation of transgenic plants). Most of the transgenic lines did not show any macroscopic difference compared to the untransformed ones, however three lines produced flowers with anomalies in the androecia (Figure 1).

Normally the tomato anthers form a sort of cone that surrounds the distal part of the style (Figure 1A, 1D). In the anomalous transgenic lines the anthers did not form a regular cone and appeared more (Figure 1B, 1E) or less (Figure 1C) splayed out. Such morphological anomaly suggested that also the anther functionality might have been affected, hence pollen viability assays were performed for the three different lines showing various degrees of splayed out stamens. As it can be seen from Table 1, all three transgenic lines exhibited a highly reduced pollen viability compared to untransformed plants, with line #16 having an extremely low amount (16%) of viable pollen. In accordance with the above data, all the three transgenic lines produced a significantly reduced amount of seeds per fruit, with line #16 yielding only seedless fruits (Table 1). Since the line with the strongest phenotype (i.e. #16) produced seedless fruits and the very little seeds produced by the other two lines were poorly viable, the subsequent molecular characterization could be carried out only with the primary transformants.

**Table 1:** **Pollen viability assay and mean number of seeds per fruit in wild-type and transgenic lines**

**Plant lines**	**Pollen viability** ^**a**^	**Seeds per fruit** ^**b**^
wild-type#1	98%	28 ± 6.5
wild-type#2	97%	26.7 ± 6.7
35S:TM8#11	25%	**5.8 ± 3.4**
35S:TM8#12	32%	**9.3 ± 4.2**
35S:TM8#16	16%	**0 ± 0**
35S:TM8:SRDX#1	97%	**0 ± 0**
35S:TM8:SRDX#2	96%	**0 ± 0**
35S:TM8:SRDX#6	96%	**0 ± 0**

^a^Pollen viability assayed according to the MTT test.

^b^Mean number ± standard deviations, Values in boldface are significantly different by Student’s t test from wild-type (P <0.05).

### Molecular characterization of plants over-expressing the *TM8* gene

**Figure 2:**
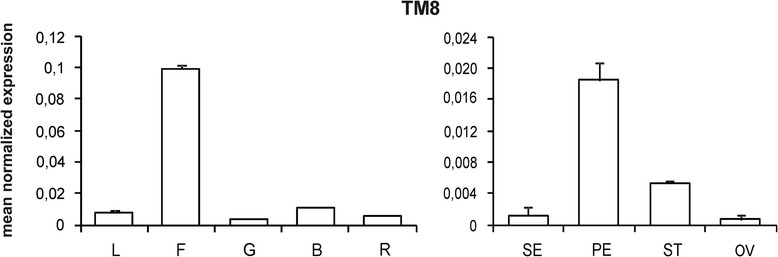
***TM8*** **gene expression pattern.** Relative expression profiles of the *TM8* gene in leaf (L), flower (F), fruits at different developmental stages: green (G), breaker (B), red (R) and flower organs: sepals (SE), petals (PE), stamens (ST) and ovaries (OV). Expression data (means of the normalized expression) were obtained by real-time PCR analyses. Bars are the standard deviations from the means.

**Figure 3:**
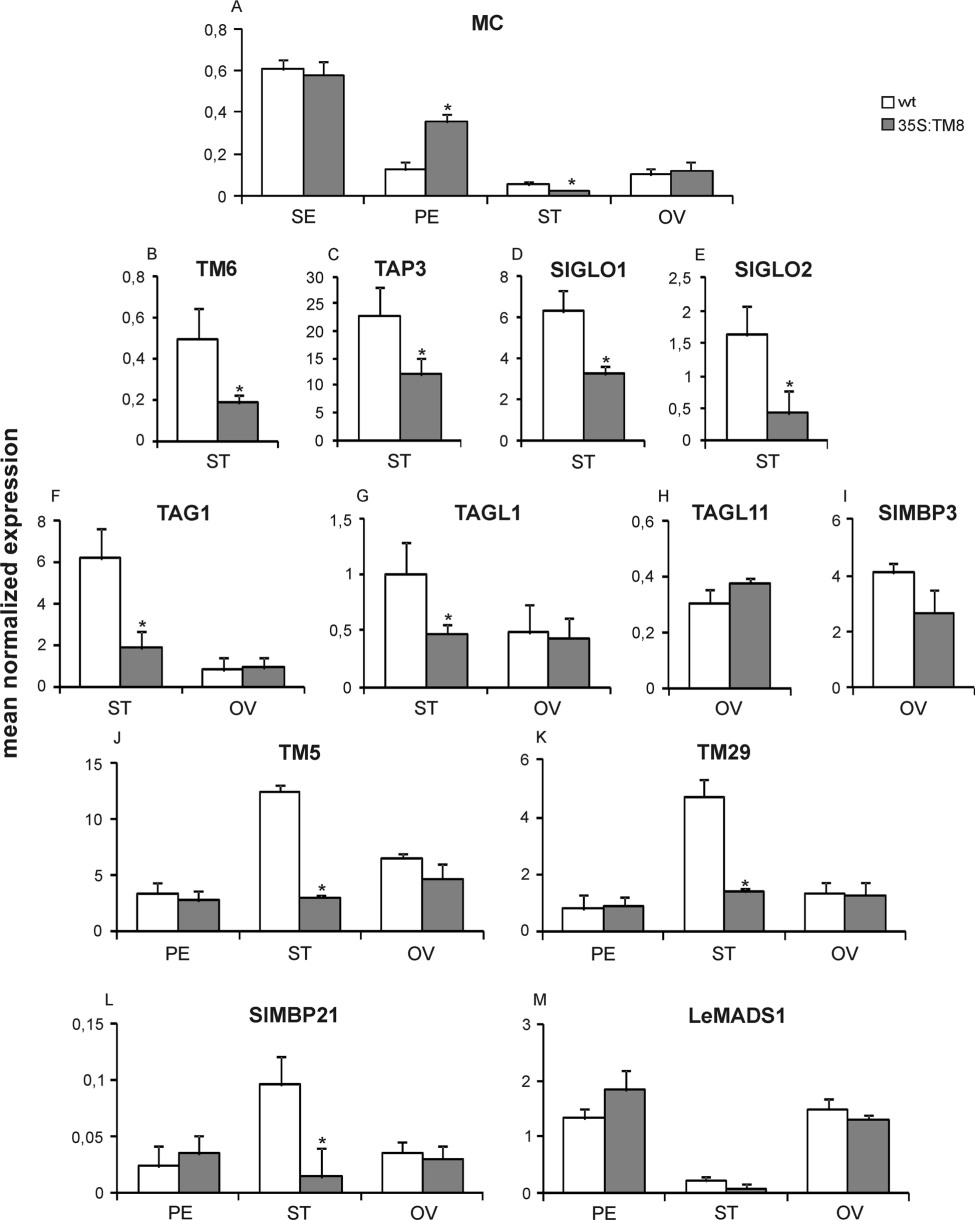
**Relative expression of MADS-box genes in** ***35S:TM8*** **plants.** Panels from **A** to **M** show the relative expression of MADS-box transcription factor encoding genes in wild-type (white) and *TM8* over-expressing (grey) flower organs: (SE), petals (PE), stamens (ST) and ovaries (OV). Each panel incorporates the name of the analyzed gene. Expression data (means of the normalized expression) were obtained by real-time PCR analyses. Values represent the mean of three different transgenic lines and two untransformed plants. Bars are the standard deviations from the means. Asterisks indicate values significantly different by Student’s t test from the control (P <0.05).

In untransformed flowers the *TM8* gene is generally expressed at very low levels in all four whorls with the highest transcript amount being found in petals, followed by anthers, sepals and ovaries (Figure 2). Analyses were carried out on three different transgenic lines and two untransformed plants (Additional file 2), and all the values shown here represent means of the three transgenic and the two untransformed plants, respectively. The only evident phenotypic effects in the *TM8* over-expressing plants were those found in the stamens (i.e. whorl 3), accordingly the expression profile of MADS-box genes normally expressed in this whorl was studied. Four genes [i.e. *TM6*, *TAP3*(*TOMATO APETALA3*), *SlGLO1* and *SlGLO2* (*Solanum lycopersicon GLOBOSA 1 and 2*)] are known in tomato that belong to the B class [3,11,12]. The over-expression of TM8 repressed the expression of the four genes in stamens (Figure 3B, C, D, E), while in petals their expression appeared increased (Additional file 3).

Considering the altered expression of the B-function genes in petals, we examined also the expression of *MACROCALYX* (*MC*) which is supposed to be an A-function gene in tomato [5,13], and should therefore be expressed in both petals and sepals according to the ABC model of Arabidopsis flower development [1]. As expected, *MC* was expressed in both sepals and petals, however transcripts were observed also in anthers and ovaries (Figure 3A). Actually, sepals had the highest transcript amount followed by petals, ovaries and anthers, respectively. In the transgenic flowers *MC* showed unchanged expression levels in sepals and ovaries, while the gene transcripts appeared increased in petals and decreased in the splayed out anthers.

*SEPALLATA* genes constitute the E function genes and are very important for the formation of all flower whorls since SEP proteins form high order complexes with the ABC transcription factors. In tomato five *SEP* genes are found, *TM5*, *TM29*, *SlMBP21* (*Solanum lycopersicon MADS-box PROTEIN 21*), *LeMADS1* (*Lycopersicon esculentum MADS1*) and *RIN* (*RIPENING-INHIBITOR*) [3,13-15], therefore also their expression was analyzed in the *TM8* over-expressing plants. Interestingly, the *TM5*, *TM29* and *SlMBP21* genes showed a highly reduced expression in the splayed out anthers compared to the control ones (Figure 3J, K, L). No apparent difference between transgenic and untransformed plants was found in whorls 2, 3 and 4 as regards *LeMADS1* (Figure 3M) and *RIN*, the latter generally expressed at extremely low levels (data not shown).

In tomato four genes [i.e. *TAG1* (*TOMATO AGAMOUS 1*), *TAGL1* (*TOMATO AGAMOUS-LIKE 1*), *TAGL11* (*TOMATO AGAMOUS-LIKE 11*) and *SLMBP3* (*Solanum lycopersicon MADS-box PROTEIN 3*)] form the *AGAMOUS* subfamily [3,15,16] although the genuine C function is represented by the *TAG1* and *TAGL1* genes while the other two represent the D function genes. Interestingly, no expression difference was found for the C function genes in the transgenic pistils while a significantly decreased expression was found in the transgenic splayed out stamens (Figure 3F,G). As regards the *TAGL11* and *SLMBP3* genes, they were expressed at high levels in ovaries and no difference was observed between *TM8* over-expressing and untransformed plants (Figure 3H,I).

### Phenotypic characterization of plants expressing the *TM8:SRDX* chimeric repressor gene

**Figure 4:**
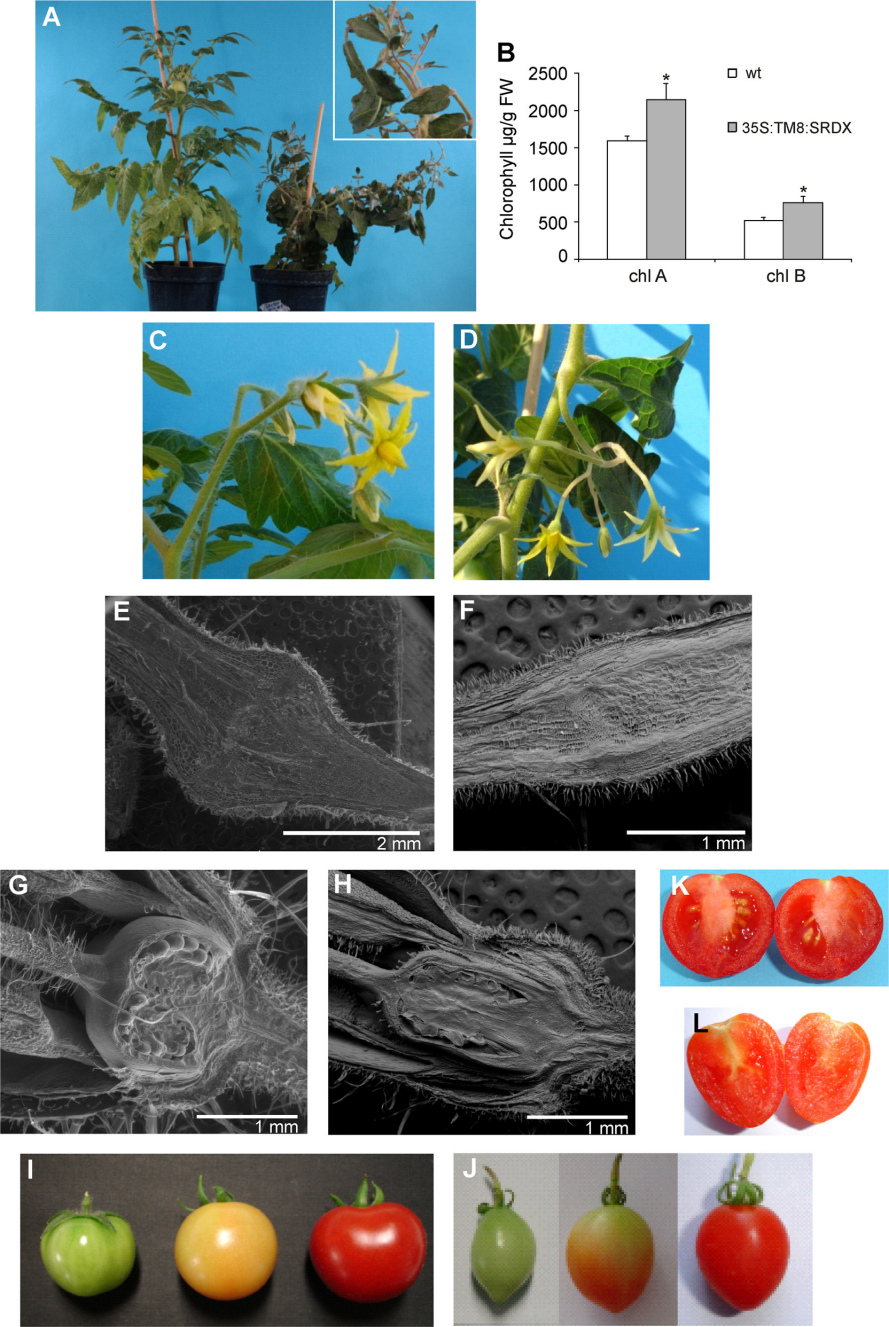
***35S:TM8:SRDX*** **plant phenotype. (A)** Wild-type tomato plant (left) and plant of the line *35S:TM8:SRDX*#2 (right) over-expressing *TM8:SRDX* and having a marked leaf epinasty (see insert). **(B)** Chlorophyll A and B content in wild-type (white) and *35S:TM8:SRDX* (grey) leaves. Values represent the mean of three different transgenic lines and two untransformed plants. Bars are the standard deviations from the means. Asterisks indicate values significantly different by Student’s t test from the control (P <0.05). Wild-type tomato inflorescence **(C)** and inflorescence of the line *35S:TM8:SRDX*#6 **(D)**, the latter having longer flower peduncles. ESEM (environmental scanning electron microscopy) pictures of a wild-type flower abscission zone **(E)** and of the anomalous flower abscission zone of the line *35S:TM8:SRDX*#2 **(F)**. ESEM pictures of a wild-type ovary **(G)** and of the oblong ovary of the line *35S:TM8:SRDX*#2 **(H)**. Wild-type fruits **(I, K)** and fruits of the line *35S:TM8:SRDX*#6 having an oblong morphology **(J)** and bearing no seeds **(L)**.

The expression of the *35S:TM8:SRDX* construct does actually express the gene in the form of a dominant repressor [8], and this technique has recently been used in tomato to study the role of the *TAGL1* MADS-box gene in fruits [17] but also of the Sl-ERF.B3 gene [18]. 15 independent transgenic lines, as judged on the basis of PCR assays carried out on genomic DNA, were obtained and most of them showed visible alterations of the foliage morphology (Figure 4A). In particular, a marked leaf epinasty was evident starting from very young seedlings. Moreover, in all lines the transgenic leaves exhibited a darker green color compared to the untransformed ones, and this was confirmed by measurements of chlorophyll content (Figure 4B).

Flower and fruit peduncles appeared longer compared to untransformed ones (Figure 4C,D), and the abscission zone did not show a normal organization even when observed by ESEM microscopy (Figure 4E,F). Stamens looked like the wild-type ones, while the fourth whorl of the transgenic plants appeared anomalous. In particular, ovaries and fruits had an oblong form instead of being roundish (Figure 4G,H,I,J), and all the transgenic lines yielded seedless fruits (Figure 4K,L and Table 1) therefore all the subsequent analyses had to be carried out using the primary transformants.

### Molecular characterization of plants expressing the *TM8:SRDX* chimeric repressor gene

Analyses were carried out on three different transgenic lines and two untransformed plants, and all the values shown here represent means of the three transgenic and the two untransformed plants respectively. As assessed by real time PCR (Additional file 4) the chimeric repressor transgene was expressed in the transformed plants, therefore the presence of an anomalous phenotype, different from the one induced by the gene over-expression, can be ascribed to the expression of the chimeric repressor.

**Figure 5:**
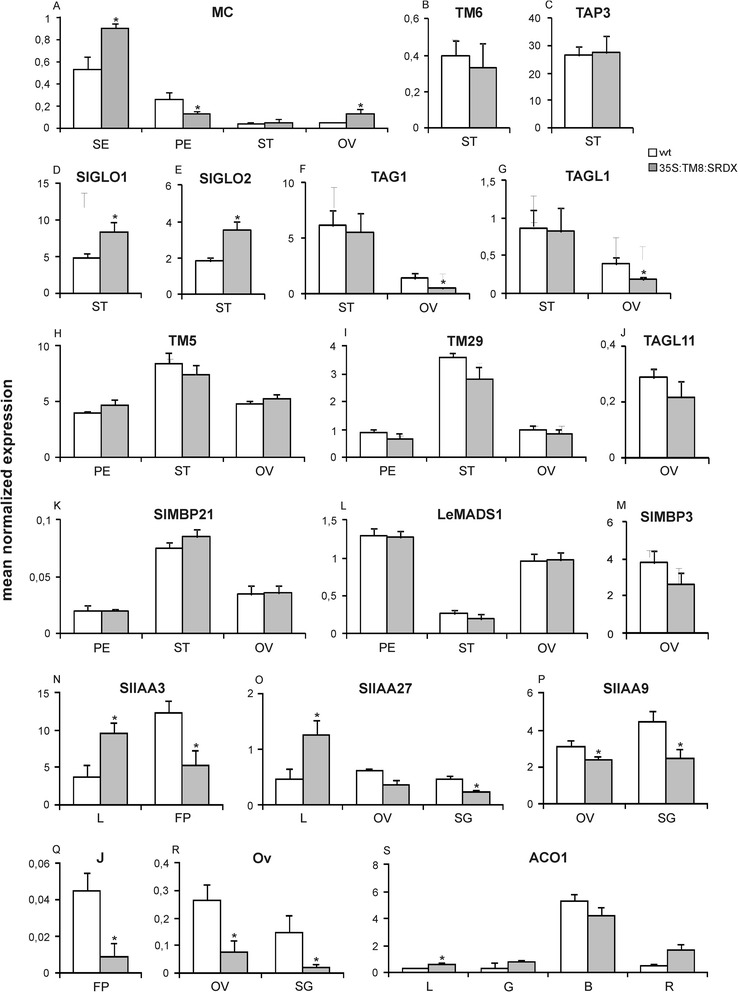
**Relative expression of transcription factor encoding genes in** ***35S:TM8*** **plants.** Relative expression of different genes in wild-type (white) and *TM8:SRDX* over-expressing (grey) tissues: sepals (SE), petals (PE), stamens (ST), ovaries (OV), flower peduncles (FP), leaf (L), and fruits at different stages of development [small green (SG), mature green (G), breaker (B) and red (R)]. Panels from **A** to **M** show the expression of MADS-box transcription factor encoding genes and each panel incorporates the name of the examined gene. Panels from **N** to **O** show the expression of genes coding for different Aux/IAA proteins whose specific name is reported in its dedicated panel. Finally, panel **Q**, **R** and **S** show the expression of the *JOINTLESS* (J), *OVATE* (OV) and *ACO1* genes, respectively. Expression data (means of the normalized expression) were obtained by real-time PCR analyses. Values represent the mean of three different transgenic lines and two untransformed plants. Bars are the standard deviations from the means. Asterisks indicate values significantly different by Student’s t test from the control (P <0.05).

It is known that in tomato the *JOINTLESS* (*J*) MADS-box gene controls the correct formation of the abscission zone in the flower and fruit peduncle [19]. Since in our plants such abscission zone did not show a correct organization, the expression of *JOINTLESS* was analyzed in these abnormal zones and the transcript amount of this gene resulted significantly reduced compared to the untransformed ones (Figure 5Q).

**Figure 6:**
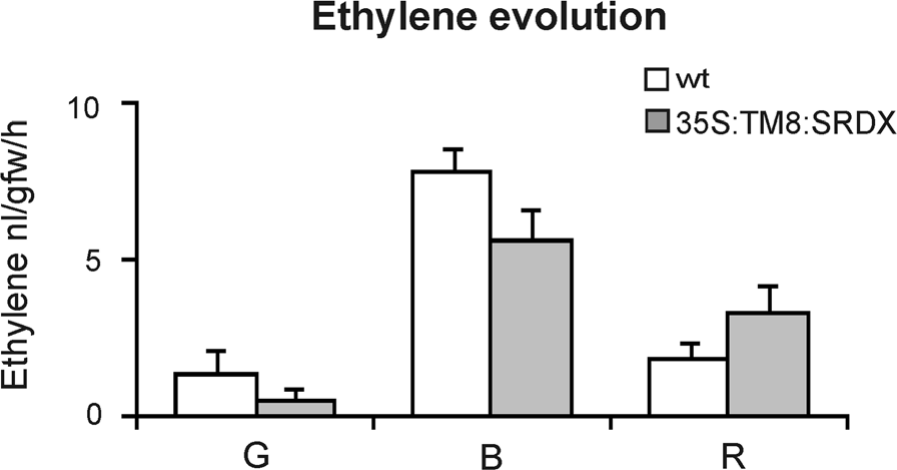
**Ethylene evolution.** Ethylene evolution in wild-type (white) and *TM8:SRDX* over-expressing (grey) fruits: green (G), breaker (B) and red (R). Values represent the mean of three different transgenic lines and two untransformed plants. Bars are the standard deviations from the means.

The marked leaf epinasty suggested at first sight an over-production of ethylene by those leaves, yet, in spite of an increased expression of the *ACO1* gene (Figure 5S), which is known to be involved in ethylene biosynthesis in tomato [20], no ethylene production could be measured under our experimental conditions. Probably in leaves the production of ethylene was below the sensitivity threshold of the used instrument since the hormone could be measured in the TM8:SRDX fruits where it turned out to be produced in amount comparable to that of the untransformed ones (Figure 6).

In dark-grown seedlings it is known that in the ethylene-induced hook formation a role is played also by auxin, and an interaction between the two hormones has been shown also for leaf epinasty [21]. In order to understand whether auxin might be involved also in the leaf epinasty observed in our transgenic plants it was decided to study the expression of those genes coding for Aux/IAA proteins whose role has already been clarified.

The *SlIAA3*(*Solanum lycopersicon IAA 3*) gene has expression regulated by both auxin and ethylene [22]. The down-regulation of this gene reduced the ethylene-induced leaf epinasty, thus suggesting a direct relation between expression of the gene and rate of epinasty [22]. In the transgenic plants with strong epinasty the expression of this gene appeared significantly increased in the leaves while it was reduced in the elongated flower peduncles (Figure 5N).

Recently, the gene *SlIAA27*(*Solanum lycopersicon IAA27*) coding for another Aux/IAA protein has been characterized [23]. In particular, it has been shown that its silencing leads to the formation of elongated fruits that look like those produced by the *35S:TM8:SRDX* tomato plants. Moreover, the *SlIAA27* silenced plants had leaves with a reduced chlorophyll content. Interestingly, in our transgenic leaves with increased chlorophyll content the *SlIAA27* gene shows an increased expression, while in the elongated fruits the transcript amount appears reduced compared to that of the untransformed fruits (Figure 5O).

Regarding *SlIAA9* (*Solanum lycopersicon IAA9*), its down-regulation led to the formation of parthenocarpic fruits [24] similarly to what observed in the*TM8:SRDX* expressing plants. Interestingly, in the latter plants the *SlIAA9* gene had a significantly reduced expression in ovaries and very young fruits (10 days after anthesis), that is when seed set is supposed to occur (Figure 5P).

The expression of various MADS-box genes involved in the formation of the reproductive structures was studied in the *TM8:SRDX* expressing plants. Of the four known tomato B class genes, *TM6* and *TAP3* did not show any significantly modified expression, while both *SlGLO1* and *SlGLO2* had a significantly increased expression both in stamens (Figure 5B,C,D,E) and in petals (Additional file 5). The expression of the *MC* gene was unaffected in anthers but not in the other three whorls. The transcript amount appeared increased in sepals and decreased in petals, while in the anomalous ovaries *MC* had significantly increased expression levels (Figure 5A). Of the four genes belonging to the *AGAMOUS* subfamily, *TAG1* and *TAGL1* (the C class genes proper) showed a decreased expression in ovaries and no variation in stamens (Figure 5F,G), while no variations at all was found in these two whorls for *TAGL11* and *SLMBP3* (Figure 5J,M). Finally, the five *SEPALLATA* genes did not show any significantly changed expression in whorls 2, 3 and 4 but for a slightly decreased expression of *TM29* in the sole transgenic anthers (Figure 5H,I,K,L).

The gene *OVATE* is known in tomato for being involved in the determination of the fruit shape, and its reduced expression could be related to a change in the fruit morphology [25]. The *TM8:SRDX* expressing plants produced elongated ovaries and fruits and the *OVATE* transcripts were significantly reduced in both ovaries and very small fruits (10 days after anthesis) which are the developmental stages most relevant for *OVATE* expression and the establishment of the fruit shape (Figure 5R).

Finally, in the elongated fruits both the production of ethylene and the expression of the *ACO1* gene were basically comparable to those of the untransformed fruits, thus confirming that the oblong fruits can undergo a normal ripening process (Figure 5S and Figure 6).

### MADS-box protein interactions

Given the notable involvement of *TM8* in the development of the tomato reproductive structures, we checked the possible ability of the TM8 protein to interact with other MADS-box transcription factors involved in the development of these structures. To this aim we carried out a large set of tests by means of the yeast two hybrid technique which is a methodology widely used to evaluate the capacity of MADS transcription factors to hetero- and/or homo-dimerize *in vitro* [26,27].

TM8 was not able to form either homo-dimers or complexes with any of the four tomato B type MADS-box transcription factors (TM6, TAP3, SlGLO1 and SlGLO2). The same incapacity to form hetero-dimers was observed when TM8 was assayed with either JOINTLESS or the three SEPALLATA-like proteins (i.e. TM5, TM29 and SlMBP21) whose cognate genes had shown a markedly affected expression in the flowers with altered *TM8* expression (Table 2). Similarly, TM8 did not hetero-dimerize with either TAG1 or TAGL1, two tomato C type MADS-box proteins. Matter of fact, the TM8-TAG1 complex could very weakly promote the transcription of just one reporter gene (*ADE2*), whilst *HIS3* was not transcribed. Moreover, the weak *ADE2* activation was only observed when using TM8 as bait and TAG1 as prey, therefore these results suggest that this interaction is not solid. Interestingly, our yeast two-hybrid assays revealed that TM8 can hetero-dimerize with MC (Table 2). The TM8-MC interaction is quite solid since it was detected both with TM8 as the bait and when MC was the bait protein. Moreover, in both cases the TM8-MC complex was able to activate the transcription of the two reporter genes (*HIS3* and *ADE2*, see Table 2).

**Table 2:** **Protein interactions in yeast**

	**-W-L** ^**a**^	**-W-L-H +2 mM 3AT** ^**b**^	**-W-L-H +5 mM 3AT** ^**c**^	**-W-L-A** ^**d**^
TM8-BD/AD	++	--	--	--
TM8-BD/TM8-AD	++	--	--	--
BD/TM8-AD	++	--	--	--
TM8-BD/MC-AD	++	++	++	++
BD/MC-AD	++	--	--	--
MC-BD/TM8-AD	++	++	+	+
MC-BD/AD	++	--	--	--
TM8-BD/TM6-AD	++	--	--	--
BD/TM6-AD	++	--	--	--
TM6-BD/TM8-AD	++	--	--	--
TM6-BD/AD	++	--	--	--
TM8-BD/TAP3-AD	++	--	--	--
BD/TAP3-AD	++	--	--	--
TAP3-BD/TM8-AD	++	--	--	--
TAP3-BD/AD	++	--	--	--
TM8-BD/SlGLO1-AD	++	--	--	--
BD/SlGLO1-AD	++	--	--	--
SlGLO1-BD/TM8-AD	++	--	--	--
SlGLO1-BD/AD	++	--	--	--
TM8-BD/SlGLO2-AD	++	--	--	--
BD/SlGLO2-AD	++	--	--	--
SlGLO2-BD/TM8-AD	++	--	--	--
SlGLO2-BD/AD	++	--	--	--
TM8-BD/TAG-AD	++	--	--	+
BD/TAG-AD	++	--	--	--
TAG-BD/TM8-AD	++	--	--	--
TAG-BD/TM8-AD	++	--	--	--
TM8-AD/TAGL1-BD	++	--	--	--
AD/ TAGL1-BD	++	--	--	--
TM8-BD/TM5-AD	++	--	--	--
BD/TM5-AD	++	--	--	--
TM5-BD/TM8-AD	++	--	--	--
TM5-BD/AD	++	--	--	--
TM-8BD/TM29-AD	++	--	--	--
BD/TM29-AD	++	--	--	--
TM-29BD/TM8-AD	++	--	--	--
TM-29BD/AD	++	--	--	--
TM8-BD/SlMBP21-AD	++	--	--	--
BD/SlMBP21-AD	++	--	--	--
SlMBP21-BD/TM8-AD	++	--	--	--
SlMBP21-BD/AD	++	--	--	--
TM8BD/J-AD	++	--	--	--
8BD/J-AD	++	--	--	--

^a^Media lacking either adenine or histidine (supplemented with *3-AT,* a competitive inhibitor of the HIS3 enzyme.

^b^-W-L YSD media lacking tryptophan and leucine.

^c^W-L – H YSD media lacking tryptophan, leucine and histidine.

^d^-W-L – A YSD media lacking tryptophan, leucine and adenine.

TM8 has been tested for its ability to form dimers with several tomato MADS-box proteins. Interactions have been assayed using TM8 both as bait and as prey. All bait and prey chimeric proteins have been controlled, then they have been transformed with the corresponding empty vector in order to exclude auto-activation problems. Yeast transformations have been repeated twice and each time at least four yeast colonies containing a bait and prey plasmids have been tested for their ability to grow in selective media.

## Discussion

Transgenic tomato plants with altered expression of the gene were prepared in order to carry out a functional characterization of *TM8*. In particular, in the *TM8* over-expressing plants macroscopic anomalies were found in whorl 3, and they basically consisted of splayed out stamens with poorly viable pollen. Regarding MADS-box genes, splayed out and sterile stamens were first reported by Pnueli *et al*. [28] in antisense tomato plants with down-regulated expression of *TM5* which is an E-function gene. In these plants other MADS-box genes, *TM6* and *TAG1* among them, were unaffected in their expression. Also Ampomah-Dwamena *et al*. [14] obtained splayed out and sterile stamens in tomato plants where *TM29*, another E-function gene, had been ectopically down-regulated, and such down-regulation did not affect the expression of the *TM5* and *TAG1* MADS-box genes. Finally, splayed out stamens were found in tomato plants with either decreased or missing expression of various B-function genes [12,29].

The above findings are particularly interesting because they show that in the same species both the morphology and the functionality of stamens could be affected in an apparently similar manner by a decreased expression of either one or another of different types of MADS-box genes. Therefore, since the down-regulation of a single gene could cause the same anomalous phenotype, it appears that in tomato the protein products of both the B-function genes and the two E-function genes *TM5* and TM29 interdependently participate in the process that leads to the differentiation of the third whorl.

The results obtained in this work add further complexity to the molecular network involved in the differentiation of whorl 3 in tomato. In fact, besides the four B-function and the two E-function genes already mentioned, also *SlMBP21*, another E-function gene, and both *TAG1* and *TAGL1*, two C-function genes, and *MACROCALYX*, a putative A-function gene, appeared to be significantly down-regulated in the anomalous splayed out stamens produced by the *TM8* over-expressing tomato plants. Since all the above genes had significantly reduced expression in the anomalous stamens, this finding suggests that in whorl 3 there must be a specific combination and dose equilibrium of various MADS-box proteins in order to have a correct differentiation of stamens. In petals (whorl 2) all the four B-function genes and *MACROCALYX* appeared significantly up-regulated in the *TM8* over-expressing petals. On the contrary, a mixed situation was found in the *TM8:SRDX* expressing petals: the *SlGLO1* and *SlGLO2* genes appeared similarly up-regulated and the *TM6* and *TAP3* genes showed no variations in expression, while *MACROCALYX* showed a decreased expression. Since no significant morphologic difference could be evidenced in petals of both types of transgenic plants, it appears that the observed changes in the expression of both *MACROCALYX* and the B-function genes is not sufficient for significantly altering the petal morphology.

However, the data regarding the expression of *MC* in our transgenic plants suggest that this gene must play some role in the differentiation of the whole tomato flower. In fact, even though *MC* is considered a putative A-function gene, its expression in the fully differentiated flowers was not restricted to the first two whorls, as one would expect on the basis of the canonical ABC model [1]. On the contrary, *MACROCALYX* was expressed in all 4 whorls, and had a significantly changed expression in both the splayed out anthers and the anomalous ovaries, respectively.

The identity of flower organs is specified by various MIKC MADS-box transcription factors which act in a combinatorial manner [1]. The molecular networks formed by these proteins have been extensively explored using yeast two hybrid assays [26,30]. Such studies have been performed also in tomato [12,15,29], however the possible interactions of the TM8 protein with other MADS-box transcription factors was never examined. We therefore decided to use this technique to identify those tomato MADS box proteins able to form heterodimers with TM8.

Unlike Arabidopsis, tomato has two AP3-like proteins, TAP3 and TM6 [3,11], and two PI-like proteins, SlGLO1 and SlGLO2 [12], which represent the B-class function. None of the B type MADS box proteins was able to form dimers with TM8 in our yeast two hybrid assays. Yeast two hybrid assays also excluded that TM8 is able to homo-dimerize. TM8 did not interact with TAGL1 and TAG1 either. Regarding TAG1, we recorded a weak activation of the *ADE2* reporter gene, but we were not able to observe growth on media lacking histidine, which suggests that *HIS3* was not activated. Therefore it appears unlikely that TM8 and TAG1 may form dimers *in vivo*.

TM8 did not physically interact in yeast with those tomato SEPALLATA-like MADS box protein [TM5, TM29 and SlMP21 [31]] that had altered expression patterns in our transgenic plants, and also with JOINTLESS [19]. Interestingly, TM8 was able to interact in yeast with MACROCALIX, and the dimer TM8-MC could promote the transcription of both reporter genes, *ADE2* and *HIS3*. This confirms that the chimeric TM8 protein used for yeast assays is properly folded.

Twenty years after the proposal of the ABC model, a modification was introduced by Causier *et al*. [32] in the review “Floral organ identity: 20 years of ABCs” to account for the absence of an A-function in most plant species. Schwarz-Sommer and co-workers introduced a new (A)-function [33] important to define the floral meristem identity and to produce the sepals that are considered as the ground state of floral organs. Our data seem to suggest that this model might apply also to the tomato flower, and that the activity of the TM8 protein might be mediated by interactions with the MACROCALYX protein.

The over-expression of the *TM8:SRDX* repressor chimera had macroscopic effects on both reproductive and vegetative structures. Although it has been shown in tomato [5] and in two Gymnosperms [34] that TM8-like genes are expressed also in leaves, the latter finding was unexpected because Lifschitz *et al*. [7] had reported anomalies only for the reproductive structures in their *TM8* antisense tomato plants, while in our transgenic plants the leaves showed a marked epinasty and were greener compared to the untransformed ones. Also the flower peduncles were different compared to wild-types since they did not differentiate a correct abscission zone. To the latter purpose, it is known that a correct expression of the MADS-box gene *JOINTLESS* is necessary for the differentiation of a normal abscission zone in the tomato flower peduncle [25] and, as expected, in the anomalous abscission zones of the *TM8:SRDX* flower peduncles also the expression of *JOINTLESS* appeared significantly reduced, in agreement with the defective abscission zones.

In plants the physiological activity of a given hormone may also depend on its interactions with other hormones present in the same tissue, and this has been shown several times for ethylene and auxin [35,36]. In tomato it was demonstrated that *SlIAA3*, a gene coding for an Aux/IAA protein, can be positively regulated by both auxin and ethylene, and antisense tomato plants for this gene had a reduced epinastic response compared to wild-type ones when treated with exogenous ethylene [36]. Interestingly, in the *TM8:SRDX* expressing plants the epinastic leaves had a significantly increased expression of the *SlIAA3* gene, while a significantly reduced transcript amount was found in the lengthened flower peduncles. Therefore, the inability to measure the ethylene produced by the epinastic leaves might simply reflect the need for ethylene to just activate the expression of the *SlIAA3*gene, therefore the hormone had not to be produced in enormous amounts. Suggestions about a possible auxin involvement came also from other phenotypic characteristics of the *35S:TM8:SRDX* plants, like the elongated fruits, their parthenocarpy and the deep green color of the foliage. In fact, in these anomalous situations other genes involved in the signal transduction pathway of auxin showed an expression that was altered as expected on the basis of their demonstrated function [22-24]. However, the possible connection between TM8 and auxin remains elusive.

As regards the phenotypic anomalies of the reproductive structures observed in the *TM8:SRDX* plants, they appeared to affect only whorl 4. In particular, all the fruits were parthenocarpic, a characteristics already described by Lifschitz *et al*. [7] for their *TM8* antisense tomato plants. On the contrary, the stamens had a normal appearance and the pollen viability was comparable with that of the wild-types, therefore the anomaly was evidently due to problems in the carpel whorl. Actually, transgenic ovaries had an elongated shape that was maintained till the end of their development so that also ripe fruits had an ellipsoidal shape instead of being roundish like the untransformed ones. The shape of tomato fruits is under the control of various genes [37], in particular a low expression of the *OVATE* gene has been shown to be responsible for the formation of pear-shaped tomatoes [25]. Recently, Rodriguez *et al*. [37] evidenced that *OVATE* may also be involved in the formation of ellipsoidal tomatoes, which appears to be the case also for the*35S:TM8:SRDX* fruits since the *OVATE* gene had a significantly decreased expression in ovaries and very young fruits, that is when the fruit shape is established.

The C-function *TAG1* gene was shown by Pnueli *et al*. [38] to be expressed in stamens and carpels, and to be of basic importance for a correct differentiation of these two organs. In particular, they found that a down-regulated expression of the gene caused the appearance of relevant malformations, among which both male and female sterility were reported. In tomato *TAG1* and *TAGL1* are the genuine C function genes while *TAGL11* and *SlMBP3* are D-function genes. It is interesting to note that the expression of both *TAG1* and *TAGL1* was consistent with the role played by them during the differentiation of reproductive structures. In the *TM8* over-expressing plants the two genes had significantly decreased expression in the anomalous stamens but not in the normal ovaries, on the contrary in the *TM8:SRDX* expressing plants the two genes had normal expression levels in stamens and significantly reduced expression levels in the anomalous ovaries. Since in the latter ovaries both the D-function and the E-function genes did not show any significantly varied expression compared to wild-type, the above data reinforce the role played by the *TAG1* and *TAGL1* genes in the development of tomato carpels [17,38,39].

## Conclusions

Soon after its discovery, the expression profile of the *TM8* gene was studied by means of a Northern analysis and the Authors found high transcript amounts in the *anantha* floral meristem where other MADS-box genes (i.e. *TM5* and *TM6*) could not be detected. For this reason the *TM8* gene was defined as an “early” gene and the others as “late” genes along the process of flower differentiation [3]. The above pattern of expression suggests that the early *TM8* gene might, in some yet unknown way, regulate the expression of the late genes, and such an idea appears to be consistent with the results of this work, at least as far as the differentiation of whorls 3 and 4 and the expression of other MADS-box genes are concerned. It will be interesting to study the relations between the expression of *TM8* and the activity of auxin, a hormone that is known to be important for fruit set and development [40].

Recently, Gramzow et al. [41] showed that *TM8-*like genes are quite common also in Gymnosperms. Therefore, in spite of the generally low levels of expression observed for this type of gene, its involvement in the formation of the reproductive structures might be the reason for the widespread conservation of TM8-like genes in seed plants.

## Methods

### Plant material

Tomato plants (*Lycopersicon esculentum* cv. Florida Petite) were grown under standard conditions at 25°C and a 16-h photoperiod in a controlled greenhouse at the Department of Biology, University of Padua. No authorization was needed for growing the tomato plants in the above greenhouse. Seeds were obtained from the Tomato Growers Supply Company, Fort Myers, FL, USA (www.tomatogrowers.com). Flower parts [sepals (SE), petals (PE), stamens (ST), ovaries (OV)] and peduncles (FP) were collected from flowers at anthesis. Fruits were harvested at different developmental stages: small green i.e. 10 days after anthesis (SG), mature green (G), breaker (B) and ripe red (R). Fully expanded leaves (L) were also harvested. All tissues and fruit samples were frozen and stored at −80°C.

### RNA extraction and gene expression analysis

Total RNA was extracted from different tissues according to Chang *et al*. [42]. RNA yield and purity were checked by means of ultraviolet (UV) absorption spectra, whereas RNA integrity was ascertained by electrophoresis in agarose gel.

The RNA samples obtained from different tissues were converted to cDNA by means of the High-Capacity cDNA Archive Kit (Applied Biosystems, www.lifetechnologies.com), using random hexamers as primers. 3 μg of total RNA, pre-treated with 1.5 U of DNase I (Promega, www.promega.com), were used as starting template. The gene expression analysis was performed by standard real-time PCR. Primer sequences for the selected genes are listed in Additional file 6. The internal standard consisted of the actin gene. PCR was carried out with the Gene Amp 7500 Sequence Detection System (Applied Biosystems). The obtained C_T_ values were analyzed by means of the Q-gene software by averaging three independently calculated normalized expression values for each sample. Expression values are given as the mean of the normalized expression values of the triplicates, calculated according to equation (2) of the Q-gene software [43].

### Generation of transgenic plants

The constructs used to produce transgenic plants were prepared using the pBINAr_GWa plasmid obtained by cloning the GWa gateway cassette (Invitrogen, www.lifetechnologies.com) into the pBINAr vector SmaI restriction site, between the 35S CaMV promoter and a nopaline synthase (NOS) terminator [44].

To obtain the *35S:TM8* construct the *TM8* (*TOMATO MADS 8*) full length cDNA (accession number KF270624) was PCR-amplified using primers (FW 5′- CATTTGAAGAATGGGGAGAG - 3′ and RV 5′-AGGTAGCAATTGAAGCTCTG - 3′) designed on the already available *TM8* sequence (X60760) and was subsequently cloned into the pCR®8/GW/TOPO® vector (Invitrogen).

Using the Gateway LR Clonase enzyme mix (Invitrogen) the *TM8* cDNA was cloned into the pBINAr_GWa plasmid via homologous recombination.

A dominant repressor construct (*35S:TM8:SRDX*) was created by generating a translational fusion between the EAR repression domain (SRDX) [8] and the 3′ end of the *TM8* cDNA by means of an RT-PCR experiment using the primers FW 5′- CATTTGAAGAATGGGGAGAG - 3′ and RV 5′-TTTTAAGCGAAACCCAAACGGAGTTCTAGATCCAGATCGAGTCCCTTAGAAAGTAACTC-3′ (the latter containing the SRDX repression domain). Subsequently the *TM8:SRDX* amplicon was introduced into the pBINAr_GWa as described above.

The identity of the cloned cDNAs was ascertained by sequencing. DNA sequencing was performed by BMR Genomics, Padua, Italy (www.bmr-genomics.it). Sequence manipulations, analyses, and alignments were performed using the LASERGENE software package (DNASTAR, www.dnastar.com).

The resulting binary plasmids were inserted in *Agrobacterium tumefaciens* (strain LBA4404) cells that were then used to transform tomato according to Fillati *et al*. [45]. Kanamycin-resistant plants were confirmed for the presence of the transgene by means of PCR, using a primer on the cauliflower mosaic virus (CaMV) 35S promoter (5′-GGGGAATTCGGTGGCTCCTACAAATG- 3′) and a primer on the *TM8* coding sequence (for the *35S:TM8* construct: 5′- TCATCCCTTAGAAAGTAACTCACT- 3′ and for the *35S:TM8:SRDX*: 5′ - TTCTAGATCCAGATCGAGTCCCTTAG- 3′).

### Pollen viability test

Pollen viability was ascertained using the MTT [MTT: 3-(4,5-dimethylthiazolyl-2)-2,5-diphenyltetrazoliumbromide] assay according to [46]. Briefly, the anthers of ten flowers taken from the plant of interest were introduced into a solution containing MTT 1% (w/v) (Sigma-Aldrich, www.sigmaaldrich.com) and sucrose 5% (w/v). After ten minutes 1000 pollen grains per plant were counted under an optical microscope (LEICA DM5000, www.leica-microsystems.com/). Pollen grains were considered viable if they turned deep pink. Wild-type anthers incubated for 2 hours at 80°C were used as negative control.

### Microscopy analysis

Tomato tissues (flower parts and peduncles) were observed without any treatment under low-pressure conditions by means of environmental scanning electron microscopy (ESEM) at the CUGAS facilities, University of Padua (www.unipd.it/cugas/).

### Ethylene measurement

Ethylene production was determined by enclosing either the whole fruits or the leaves in jars (50–60 ml, according to need), sealed with a transparent wrapper and kept in the light at room temperature. After 1 h, 1 ml of air sample was withdrawn from each jar for the ethylene measurements. A gas chromatograph (Perkin–Elmer F17; Norwalk, Conn, www.perkinelmer.com) was used for quantifying ethylene concentrations.

### Yeast two-hybrid and ternary-trap assays

YPAD, SD, and appropriate dropout media have been described previously [47]. The yeast two-hybrid assays were performed using the yeast strain AH109 (Clontech, www.clontech.com) [48]. The pGBTKT7 vector (Clontech), carrying the GAL4 DNA-binding domain, was used to express the bait proteins, whereas the pGADT7 vector [49], carrying the GAL4 activation domain, was used to express the prey proteins. Two-hybrid protein interactions were evaluated by growing the yeast colonies at 28°C on media lacking either histidine or adenine and supplemented with different amounts of 3-amino-1,2,4-triazole (3-AT).

All the primers used to clone the tomato MADS box genes are listed in the Additional file 7.

### Accession number

The *TM8* sequence used in this work will appear in Genbank under the following accession number: KF270624.

## Additional files

Additional file 1:
***TM8*** **genomic and RT-PCR analysis.**
Additional file 2:
**Relative expression of** ***TM8*** **gene in transgenic** ***35S:TM8*** **lines.**
Additional file 3:
**Relative expression of B-type MADS-box transcription factor encoding genes in** ***35S:TM8*** **flower petals.**
Additional file 4:
**Relative expression of** ***TM8:SRDX*** **chimeric gene in transgenic** ***35S:TM8:SRDX*** **lines.**
Additional file 5:
**Relative expression of B-type MADS-box transcription factor encoding genes in** ***35S:TM8:SRDX*** **petals.**
Additional file 6:
**Sequences of the oligonucleotides used in the real-time PCR experiments.**
Additional file 7:
**Sequences of the oligonucleotides used in the yeast two hybrid experiments.**
