# Supplementary material for: Characterization of TM8, a MADS-box gene expressed in tomato flowers
Source: BMC Plant Biol. 2014 Nov 30;14:319. doi: 10.1186/s12870-014-0319-y (PMC4258831; doi:10.1186/s12870-014-0319-y)
Supplement: Additional file 1: — TM8 genomic and RT-PCR analysis. [file 12870_2014_319_MOESM1_ESM.pdf]

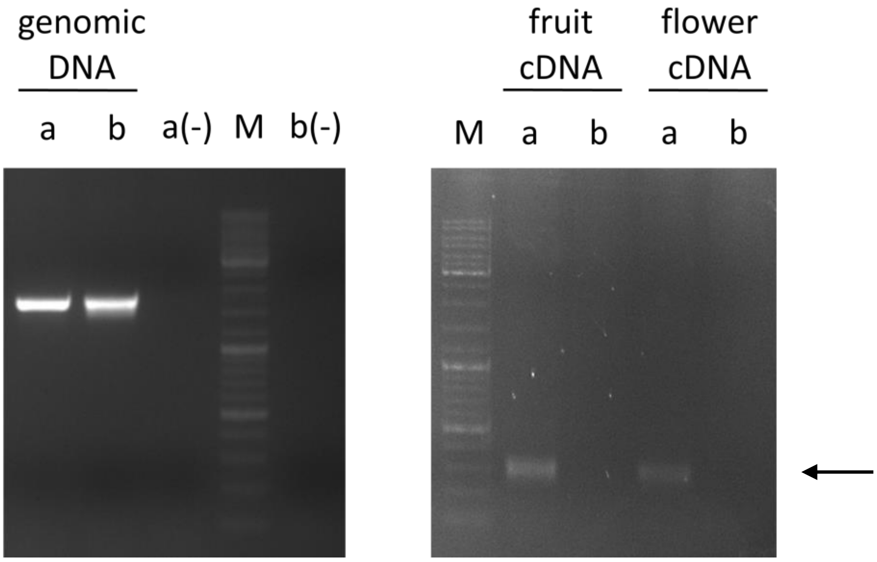

Additional file 1: PCR amplification of the *TM8* gene from genomic DNA (left panel) and from fruit and flower cDNA (right panel). In lanes 'a' the PCR products obtained by using the RT1979/RT1981 primers which discriminate the *TM8* sequence isolated in this work (KF270624); in lanes 'b' the PCR products obtained by using the RT1979/RT1980 which discriminate the X60760 TM8 sequence [3].

Gene	primer FW	primer FW sequence (5'-3')	primer RV	primer RV sequence (5'-3')
TM8	RT 1979 (*TM8* common region)	CAATACATGAACTTGAAGC	RT 1980 - X60760	TCATCCTTGGATTTGTATCG
			RT 1981 - KF270624	TCATCCCTTAGAAAGTAACTCACTT
