# Supplementary material for: Characterization of TM8, a MADS-box gene expressed in tomato flowers
Source: BMC Plant Biol. 2014 Nov 30;14:319. doi: 10.1186/s12870-014-0319-y (PMC4258831; doi:10.1186/s12870-014-0319-y)
Supplement: Additional file 2: — Relative expression of TM8 gene in transgenic 35S:TM8 lines. [file 12870_2014_319_MOESM2_ESM.pdf]

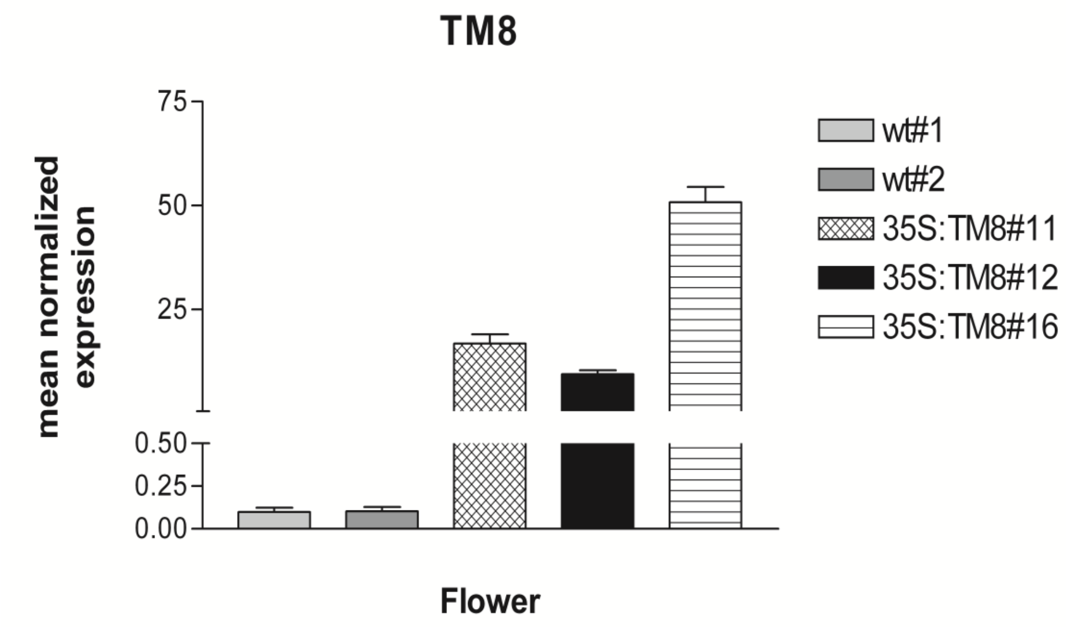

Additional file 2: Relative expression of *TM8* gene in flowers at anthesis of the two wild-type and the three *35S:TM8* lines analyzed in this work. Expression data (means of the normalized expression) were obtained by real-time PCR analyses. Bars are the standard deviations from the means.
