# Supplementary material for: Characterization of TM8, a MADS-box gene expressed in tomato flowers
Source: BMC Plant Biol. 2014 Nov 30;14:319. doi: 10.1186/s12870-014-0319-y (PMC4258831; doi:10.1186/s12870-014-0319-y)
Supplement: Additional file 3: — Relative expression of B-type MADS-box transcription factor encoding genes in 35S:TM8 flower petals. [file 12870_2014_319_MOESM3_ESM.pdf]

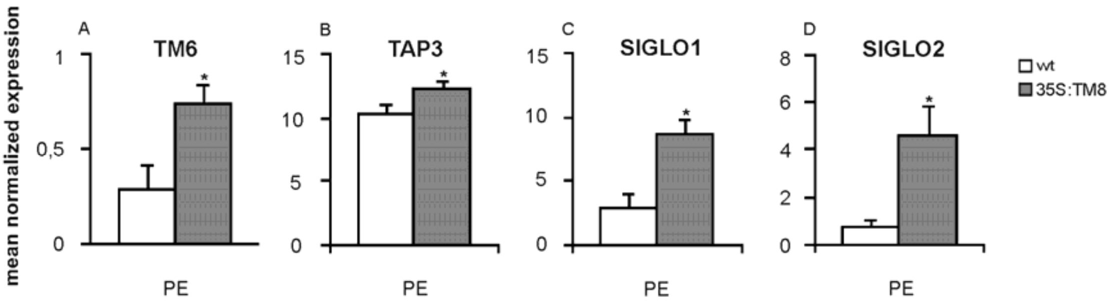

Additional file 3: Relative expression of B-type MADS-box transcription factor encoding genes in wild-type (white) and *TM8* over-expressing (grey) flower petals (PE). Expression data (means of the normalized expression) were obtained by real-time PCR analyses. Values represent the mean of three different transgenic lines and two untransformed plants. Bars are the standard deviations from the means. Asterisks indicate values significantly different by Student’s t test from the control ($P < 0.05$).
