# Supplementary material for: Characterization of TM8, a MADS-box gene expressed in tomato flowers
Source: BMC Plant Biol. 2014 Nov 30;14:319. doi: 10.1186/s12870-014-0319-y (PMC4258831; doi:10.1186/s12870-014-0319-y)
Supplement: Additional file 4: — Relative expression of TM8:SRDX chimeric gene in transgenic 35S:TM8:SRDX lines. [file 12870_2014_319_MOESM4_ESM.pdf]

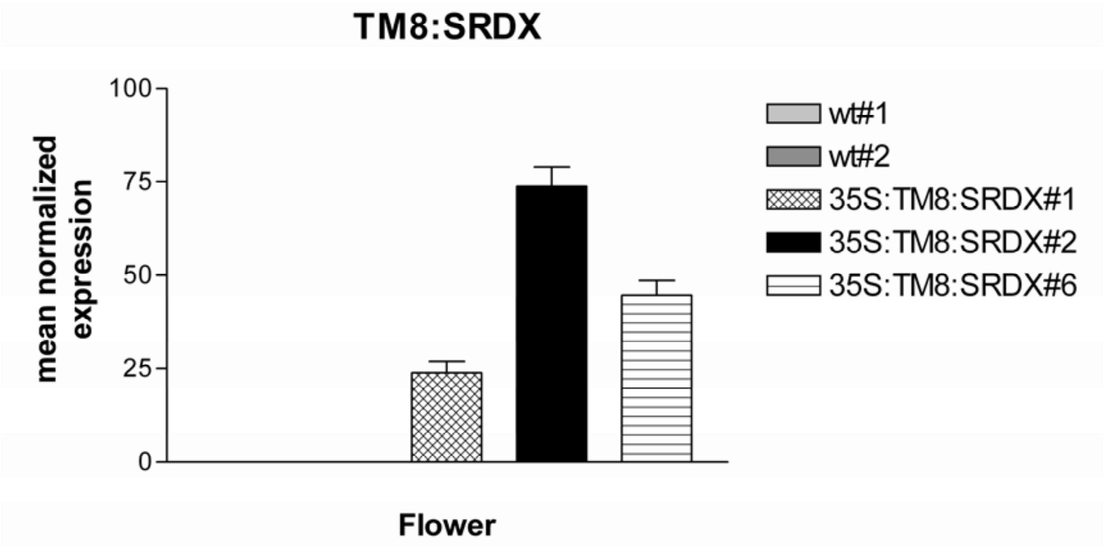

Additional file 4. Relative expression of *TM8: SRDX* chimeric gene in flowers at anthesis of two wild-type and the three *35S:TM8:SRDX* lines analyzed in this study. Expression data (means of the normalized expression) were obtained by real-time PCR analyses. Bars are the standard deviations from the means.
