# Supplementary material for: Characterization of TM8, a MADS-box gene expressed in tomato flowers
Source: BMC Plant Biol. 2014 Nov 30;14:319. doi: 10.1186/s12870-014-0319-y (PMC4258831; doi:10.1186/s12870-014-0319-y)
Supplement: Additional file 6: — Sequences of the oligonucleotides used in the real-time PCR experiments. [file 12870_2014_319_MOESM6_ESM.pdf]

Gene	primer FW name	primer FW sequence (5'-3')	primer RV name	primer RV sequence (5'-3')	Ac. number
ACO1 - 1-aminocyclopropane-1-carboxylic acid oxidase	ACO1_rtfor	TCATACAGACGCAGGAGGCA	ACO1_rtrev	GCATGGGAGGAACATCGATC	X04792
JOINTLESS	Jointless_rtfor2	AGATCTCTTGAAACTGGATTGAGC	Jointless_rtrev2	CACCTGTTGCCTTAATTTTTCAT	AF275345.3
MADS1 - Solanum lycopersicon MADS1	MADS1_rtfor	TTCGATTGGTGGGAAGATGGAG	MADS1_rtrev	GTTGCCGCTGCATTCACCTCAT	AY294329.1
MC - MACROCALYX	MC_rtfor2	GAACTGCAGAAAAAGGAAAGAGC	Mc_rtrev2	GGCATTGATGTGGTTGTAAGAGG	AF448521
OV - OVATE	SlOvate_rtfor1	AGGACCCGTACGAAGATTTCAAG	SlOvate_rtrev1	AGAACGCCTCAACTATCACTCCAT	AY140893
SlGLO1 - Solanum lycopersicon GLOBOSA 1	SlGLO1_rtfor	ATGCAAATAGAACTCAGGCACCTA	SlGLO1_rtrev	TCCTGCTCCATATTTTGAGTCTTT	XM_004245154
SlGLO2 - Solanum lycopersicon GLOBOSA 1	SlGLO1_rtfor	GGTTAAGCTCAGGCACCTCAAA	SlGLO2_rtrev	AGATTTCAGACTGCTTGGCACTG	DQ674531
SlIAA3 - Solanum lycopersicon auxin/indole-3-acetic acid 3	SlIAA3_rtfor	CAATGATCTTGAGGCAACTGAGC	SlIAA3_rtrev	TTTGGTGCAGGAGCTGAGTCTT	JN379433
SlIAA9- Solanum lycopersicon auxin/indole-3-acetic acid 9	SlIAA9_rtfor	GCTCTTCCCCTTGCACCCTTCC	SlIAA9_rtrev	TCTCCTTTGTATCACCTGCTTTCA	JN379437
SlIAA27- Solanum lycopersicon auxin/indole-3-acetic acid 27	SlIAA27_rtfor	AATGATGATGAAGGGAGAACAGGT	SlIAA27_rtrev	TGGAATCTTATCACTGGCACACTG	JN379450
SlMBP3 - Solanum lycopersicon MADS-box protein 3	SlMBP3_rtfor1	GCCAGCAGCAGGAGGACAAG	SlMBP3_rtrev1	ATGATGAGGAGGAGGCAATGGA	SGN-U585391
SlMBP21- Solanum lycopersicon MADS-box protein 21	SlMBP21_rtfor	CTTCCAGCATGATGACAACACTTG	SlMBP21_rtrev	AGATGCATCCAATTGGTCTCA	Hileman et al., 2006
Tact - Tomato actin	Tact_rtfor	AGGCACCCCTTAATCCCAAG	Tact_rtrev	AAGCACAGCCTGGATAGCAAC	AB199316
TAG1 - Tomato AGAMOUS	TAG1_rtfor	AAGGAAACTAGGAAGGGGGAAAAT	TAG1_rtrev	AACCAAAGCAACCTCAGCATCACA	AY098733
TAGL1 - Tomato AGAMOUS-like 1	TAGL1_rtfor	TCAGCCAAATTACGAAGATGC	TAGL1_rtrev	AAGCTGGAGAGGAGTTTGGTCA	NM_001247258
TAGL11 - Tomato AGAMOUS-like 11	TAGL11_rtfor1	GAGACTGAGGATTTGCACAAGAGG	TAGL11_rtrev2	TTCCATCATGTTGAGCTGTAGCAT	AY098736
TAP3 - Tomato APETALA 3	TAP3_rtfor2	TGAATTTGATGCAAGACAAGAGGA	TAP3_rtrev2	TGTTGGGTTGAAGGCGTAAGTCT	DQ674532
TM5 - Tomato MADS-box 5	TM5_rtfor2	AGCGATCACAGAGGAATCTTCTTG	TM5_rtrev2	TTCAATGCATGTTCCTTTCTCTGA	X60480
TM6 - Tomato MADS-box 6	TM6_rtfor	AGGCGAGGAACTTAGAAGAGCA	TM6_rtrev	TTGGGGTGCAATGGTTGTAGG	X60759
TM8 - Tomato MADS-box 8	TM8_rtfor1	AGGCGTATCATTTCCGAACA	TM8_rtrev1	TCATCCCTTAGAAAGTAACTCACT	KF270624
TM29 - Tomato MADS-box 29	TM29_rtfor	TCTCAATTATGGTCAGCAGCAACA	TM829_rtrev	TTTCATTCACAGCATCCAACCAG	AJ302015

Additional file 6: Sequences of the forward (FW) and reverse (RV) oligonucleotides used in the real-time PCR experiments performed in this work. In the last column the accession numbers are reported.
