# Supplementary material for: Characterization of TM8, a MADS-box gene expressed in tomato flowers
Source: BMC Plant Biol. 2014 Nov 30;14:319. doi: 10.1186/s12870-014-0319-y (PMC4258831; doi:10.1186/s12870-014-0319-y)
Supplement: Additional file 7: — Sequences of the oligonucleotides used in the yeast two hybrid experiments. [file 12870_2014_319_MOESM7_ESM.pdf]

Gene	primer FW name	primer FW sequence (5'-3')	primer RV name	primer RV sequence (5'-3')
JOINTLESS	AtP_4719	GGGGACAAGTTTGTACAAAAAAGCAGGCTcgATGAAACAAATTCTTGAGAGG	AtP_4720	GGGGACCACTTTGTACAAGAAAGCTGGGTgTTACTTTTTTTTTTCTCC
MACROCALYX	AtP_4865	GGGGACAAGTTTGTACAAAAAAGCAGGCTcgATGGGAAGAGGAAAAGTTGAATT	AtP_4866	GGGGACCACTTTGTACAAGAAAGCTGGGTgTCATAGATGTTTATTCATGTTG
SlGLO1	Atp_5341	GGGGACAAGTTTGTACAAAAAAGCAGGCTcgATGGGAAGAGGAAAGATAGAG	Atp_5342	GGGGACCACTTTGTACAAGAAAGCTGGGTgTTAGAACCTTTGGTGCAAATTAG
SlGLO2	Atp_5339	GGGGACAAGTTTGTACAAAAAAGCAGGCTcgATGGGGAGAGGTAAAATAGAG	Atp_5340	GGGGACCACTTTGTACAAGAAAGCTGGGTgTTACATTCTTTCATGTAGATTG
SlMBP21	Atp_5258	GGGGACAAGTTTGTACAAAAAAGCAGGCTcgATGGGAAGAGGAAGAGTAGAAC	Atp_5259	GGGGACCACTTTGTACAAGAAAGCTGGGTgTTAGAGCATCCACCCTGGAAT
TAG1	AtP_5099	GGGGACAAGTTTGTACAAAAAAGCAGGCTcgATGGACTTCCAAAGTGATCTAAC	AtP_5100	GGGGACCACTTTGTACAAGAAAGCTGGGTgTTAGACTAGTTGAATAGGGGGTTG
TAGL1	AtP_5097	GGGGACAAGTTTGTACAAAAAAGCAGGCTcgATGGTTTTTCCTATTAATCAGG	AtP_5098	GGGGACCACTTTGTACAAGAAAGCTGGGTgTCAGACAAGCTGGAGAGGAGTTTG
TM5	Atp_5167	GGGGACAAGTTTGTACAAAAAAGCAGGCTcgATGGGAAGGGGTAGGGTTGAGC	Atp_5168	GGGGACCACTTTGTACAAGAAAGCTGGGTgTCAAGGCAACCAGCCAGCCATG
TM8	tom1	GGGGACAAGTTTGTACAAAAAAGCAGGCTccATGGGGAGAGGAAAAGTTGAATTGA	tom2	GGGGACCACTTTGTACAAGAAAGCTGGGTcTCATCCCTTAGAAAGTAACTCACTT
TM29	Atp_5169	GGGGACAAGTTTGTACAAAAAAGCAGGCTcgATGGGTAGAGGAAGAGTTGAGC	Atp_5170	GGGGACCACTTTGTACAAGAAAGCTGGGTgTCACAGCATCCAACCAGGTATC
TAP3	AtP_4984	GGGGACAAGTTTGTACAAAAAAGCAGGCTcg ATGGCTCGTGGTAAGATCCAGATC	AtP_4985	GGGGACCACTTTGTACAAGAAAGCTGGGTg TCAACCTAGAGCAAAAGTAGTAAT

Additional file 7: Sequences of the forward (FW) and reverse (RV) oligonucleotides used in the yeast two hybrid experiments performed in this work.
